# RiboDiff: detecting changes of mRNA translation efficiency from ribosome footprints

**DOI:** 10.1093/bioinformatics/btw585

**Published:** 2016-09-14

**Authors:** Yi Zhong, Theofanis Karaletsos, Philipp Drewe, Vipin T Sreedharan, David Kuo, Kamini Singh, Hans-Guido Wendel, Gunnar Rätsch

**Affiliations:** 1 Computational Biology Program, Sloan Kettering Institute, New York, NY, USA; 2 Max Delbrück Center for Molecular Medicine, Berlin, Germany; 3 Cancer Biology Program, Sloan Kettering Institute, New York, NY, USA; 4 Department of Computer Science, ETH Zurich, Universitatsstrasse 6, Zrich, Switzerland

## Abstract

**Motivation:**

Deep sequencing based ribosome footprint profiling can provide novel insights into the regulatory mechanisms of protein translation. However, the observed ribosome profile is fundamentally confounded by transcriptional activity. In order to decipher principles of translation regulation, tools that can reliably detect changes in translation efficiency in case–control studies are needed.

**Results:**

We present a statistical framework and an analysis tool, RiboDiff, to detect genes with changes in translation efficiency across experimental treatments. RiboDiff uses generalized linear models to estimate the over-dispersion of RNA-Seq and ribosome profiling measurements separately, and performs a statistical test for differential translation efficiency using both mRNA abundance and ribosome occupancy.

**Availability and Implementation:**

RiboDiff webpage http://bioweb.me/ribodiff. Source code including scripts for preprocessing the FASTQ data are available at http://github.com/ratschlab/ribodiff.

**Supplementary information:**

Supplementary data are available at *Bioinformatics* online.

## 1 Introduction

The recently described ribosome footprinting technology (Ingolia *et al.*, 2012) allows the identification of mRNA fragments that were protected by the ribosome. It provides valuable information on ribosome occupancy and, thereby indirectly, on protein synthesis activity. This technology can be leveraged by combining the measurements from RNA-Seq estimates in order to determine a gene’s translation efficiency (TE), which is the ratio of the abundances of translated mRNA and available mRNA (Ingolia *et al.*, 2011). The normalization by mRNA abundance is designed to remove transcriptional activity as a confounder of RF abundance. The TEs in treatment/control experiments can then be compared to identify genes most affected w.r.t. translation efficiency. For instance, Thoreen *et al.* (2012) considered a ratio (fold-change) of the TEs of treatment and control. However, what these initial approaches only take into account partially is that one typically only obtains uncertain estimates of the mRNA and ribosome abundance. In particular for lowly expressed genes, the error bars for the ratio of two TE values can be large. As in proper RNA-Seq analyses, one should consider the uncertainty in these abundance measurements when testing for differential abundance. For RNA-Seq, this has been described in various ways often based on generalized linear models taking advantage of dispersion information from biological replicates (Anders *et al.*, 2012; Drewe *et al.*, 2013; Robinson *et al.*, 2010). In Wolfe *et al.* (2014) and Zhong *et al.* (2015), a way to adopt an approach for RNA-Seq analysis for this problem was described that had several conceptual and practical limitations. Here, we describe a novel statistical framework that also uses a generalized linear model to detect effects of a particular treatment on mRNA translation. Additionally, our approach accounts for the fact that two different sequencing protocols with distinct statistical characteristics are used. We compare it to the *Z*-score based approach (Thoreen *et al.*, 2012), DESeq2 (Love *et al.*, 2014) and a recently published tool Babel (Olshen *et al.*, 2013) that is based on errors-in-variables regression. Shell and Python scripts for trimming RF adaptor, aligning reads, removing rRNA contamination and counting reads are also included in the RiboDiff package.

## 2 Methods

**Fig. 1:**
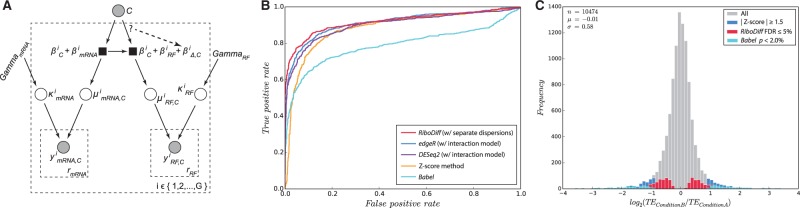
(**A**) Graphical model representing RidoDiff (Gray circle: observable variables; empty circle: unobservable variables; black square: functions; *r* denotes biological replicates; *i* denotes a gene and *G* is the number of genes). The dashed line denotes the relationship that we aim to test (see Methods for details). (**B**) Receiver operating characteristic (ROC) curve of RiboDiff (with separate dispersions), edgeR and DESeq2 (with interaction model), Z-score method and Babel on simulated data with large difference between dispersions of RF and RNA-Seq counts (see also Supplementary Fig. S-4). (**C**) Comparison of the distribution of TE ratios of genes that were detected to have a significant change in translation efficiency by RiboDiff (w/joint dispersion), *Z*-score based analysis and Babel. DESeq2 was very similar to RiboDiff (w/joint dispersion) and was omitted. Data was taken from GEO accession GSE56887 (Color version of this figure is available at *Bioinformatics* online.)

In sequencing-based ribosome footprinting, the RF read count is naturally confounded by mRNA abundance (Fig. 1A). We seek a strategy to compare RF measurements taking mRNA abundance into account in order to accurately discern the translation effect in case–control experiments. We model the vector of RNA-Seq and RF read counts $y_{\text{mRNA}}^{i}$ and $y_{\text{RF}}^{i}$, respectively, for gene *i* with Negative Binomial (NB) distributions, as described before (for instance, Love *et al.*, 2014; Drewe *et al.*, 2013; Robinson *et al.*, 2010): $y^{i}\;∼\;NB(\mu^{i},\kappa^{i}),$ where μ^*i*^ is the expected count and κ^*i*^ is the estimated dispersion across biological replicates. Here *y^i^* denotes the observed counts normalized by the library size factor (Supplementary Section A). Formulating the problem as a generalized linear model (GLM) with the logarithm as link function, we can express expectations on read counts as a function of latent quantities related to *mRNA abundance* β_*C*_ in the two conditions (C={0,1}), a quantity $\beta_{\text{RNA}}$ that relates mRNA abundance to RNA-Seq read counts, a quantity $\beta_{\text{RF}}$ that relates mRNA abundance to RF read counts and a quantity $\beta_{\Delta ,C}$ that captures the effect of the treatment on translation. In particular, the expected RNA-Seq read count $\mu_{\text{mRNA},C}^{i}$ is given by the equation $\text{log}(\mu_{\text{mRNA},C}^{i})=\beta_{C}^{i}+\beta_{\text{RNA}}^{i}$.

We assume that transcription and translation are successive cellular processing steps and that abundances are linearly related. The expected RF read count, $\mu_{\text{RF},C}^{i}$, is given by $\text{log}(\mu_{\text{RF},C}^{i})=\beta_{C}^{i}+\beta_{RF}^{i}+\beta_{\Delta ,C}^{i}$. A key point to note is that $\beta_{C}^{i}$ is revealed to be a shared parameter between the expressions governing the expected RNA-Seq and RF counts. It can be considered to be a proxy for shared transcriptional/translation activity under condition *C* in this context. Then, $\beta_{\Delta ,C}^{i}$ indicates the deviation from that activity under condition *C*, with $\beta_{\Delta ,C}^{i}=0$ for *C* = 0 and free otherwise (See Supplementary Section B for more details).

Fitting the GLM consists of learning the parameters β^*i*^ and dispersions κ^*i*^ given mRNA and RF counts for the two conditions C={0,1}. We perform alternating optimization of the parameters β^*i*^ given dispersions κ^*i*^ and the dispersion parameters κ^*i*^ given β^*i*^, similar to the EM algorithm (Supplementary Sections B and C):

$$\beta^{i}=\underset{\beta^{i}}{\text{arg}\;\text{max}}\ell_{glm}(\beta^{i}|y^{i},\kappa^{i})\;\text{and}\;\kappa^{i}=\underset{\kappa^{i}}{\text{arg}\;\text{max}}\ell_{NB}(\kappa^{i}|y^{i},\mu^{i}).$$

As experimental procedures for measuring mRNA counts and RF counts differ, we enable the estimating of separate dispersion parameters for the data sources of RNA-Seq and RF profiling to account for different characteristics (Supplementary Section E).

As in Anders *et al.* (2012), with raw dispersions estimated from previous steps, we regress all κ^*i*^ given the mean counts to obtain a mean-dispersion relationship $f(\mu )=\lambda_{1}/\mu +\lambda_{0}$. We perform empirical Bayes shrinkage (Love *et al.*, 2014) to shrink κ^*i*^ towards f(μ) to stabilize estimates (see Supplementary Section D). The proposed model in RiboDiff with a joint dispersion estimate is conceptually identical to using the following GLM design matrix protocol+condition+condition:protocol (for instance, in conjunction with edgeR or DESeq1/2).

In a treatment/control setting, we can then evaluate whether a treatment (*C* = 1) has a significant differential effect on translation efficiency compared to the control (*C* = 0). This is equivalent to determining whether the parameter $\beta_{\Delta ,1}$ differs significantly from 0 and whether the relationship denoted by the dashed arrow in Figure 1A is needed or not. We can compute significance levels based on the $\chi^{2}$ distribution by analyzing log⁡-likelihood ratios of the Null model ($\beta_{\Delta ,1}^{i}=0$) and the alternative model (βΔ,1i=0).

## 3 Results and discussion

We simulated data with different dispersions applied to mRNA and RF counts (see Supplementary Section F). We illustrate the performance of our method RiboDiff (with separate dispersion estimates) as well as Babel and the *Z*-score method. Although conceptually closely related to RiboDiff with joint dispersion estimates, we also include DESeq2 and edgeR with a GLM that includes an interaction term (GLM condition+protocol+condition:protocol) to model RNA-seq and RF counts. Figure 1B shows the receiver operating characteristic (ROC) curve for a case with large dispersion differences between RF and RNA-seq counts. RiboDiff exhibits a superior detection accuracy compared to edgeR, DESeq2, Babel and *Z*-score method, which is less pronounced when RF and RNA-Seq dispersions are more similar (see Supplementary Fig. S-4). We obtained close to identical results for RiboDiff with joint dispersion and DESeq2 with interaction term, although edgeR with the same setting is slightly better than RiboDiff with joint dispersion (data not shown). Our experiments illustrate that it can be beneficial to use the RiboDiff model with separate dispersions, in particular, when the dispersions of RF and RNA-seq data differ considerably.

We also re-analyzed previously released ribosome footprint data (GEO accession GSE56887). After multiple testing correction, RiboDiff detected 601 TE down-regulated genes and 541 up-regulated ones with FDR ≤ 0.05, which is about twice as many as reported previously. The new significant TE change set includes more than 90% genes identified in the previous study. RiboDiff is also compared to *Z*-score method and we find major differences (see Fig. 1C). Supplementary Section G provides the evidences showing that the *Z*-score based method is biased towards genes with low read count, whereas RiboDiff identifies more plausible differences. Babel identifies only very few genes with differential TE. We ran the differential test of RiboDiff on a machine with 1.7 GHz CPU and 4 GB RAM, it took 23 min of computing time to finish (10 474 genes having both mRNA and RF counts).

In summary, we propose a novel statistical model to analyze the effect of the treatment on mRNA translation and to identify genes of differential translation efficiency. A major advantage of this method is facilitating comparisons of RF abundance by taking mRNA abundance variability as a confounding factor. Moreover, RiboDiff is specifically tailored to produce robust dispersion estimates for different sequencing protocols measuring gene expression and ribosome occupancy that have different statistical properties. The described approach is statistically sound and identifies a similar set of genes from a less developed method that was used in recent work Wolfe *et al.* (2014). The release of this tool is expected to enable proper analyses of data from many future RF profiling experiments (e.g. Su *et al.*, 2015). The described model assumes that RNA-seq and RF samples are unpaired and it is future work to extend the flexibility of the tool to a broader range of experimental settings.

## Supplementary Material

Supplementary Data

## References

[btw585-B1] Anders S. et al (2012) Detecting differential usage of exons from RNA-seq data. Genome Res., 22, 2008–2017. 22722343 10.1101/gr.133744.111 PMC3460195

[btw585-B2] Drewe P. et al (2013) Accurate detection of differential RNA processing. Nucleic Acids Res., 41, 5189–5198. 23585274 10.1093/nar/gkt211 PMC3664801

[btw585-B3] Ingolia N.T. et al (2011) Ribosome profiling of mouse embryonic stem cells reveals the complexity and dynamics of mammalian proteomes. Cell, 147, 789–802. 22056041 10.1016/j.cell.2011.10.002 PMC3225288

[btw585-B4] Ingolia N.T. et al (2012) The ribosome profiling strategy for monitoring translation in vivo by deep sequencing of ribosome-protected mRNA fragments. Nat. Protoc., 7, 1534–1550. 22836135 10.1038/nprot.2012.086 PMC3535016

[btw585-B5] Love M.I. et al (2014) Moderated estimation of fold change and dispersion for RNA-seq data with deseq2. Genome Biol., 15, 550. 25516281 10.1186/s13059-014-0550-8 PMC4302049

[btw585-B6] Olshen A.B. et al (2013) Assessing gene-level translational control from ribosome profiling. Bioinformatics, 29, 2995–3002. 24048356 10.1093/bioinformatics/btt533 PMC3834798

[btw585-B7] Robinson M.D. et al (2010) edger: a bioconductor package for differential expression analysis of digital gene expression data. Bioinformatics, 26, 139–140. 19910308 10.1093/bioinformatics/btp616 PMC2796818

[btw585-B8] Su X. et al (2015) Interferon- regulates cellular metabolism and mRNA translation to potentiate macrophage activation. Nat. Immunol., 16, 838–849. 26147685 10.1038/ni.3205 PMC4509841

[btw585-B9] Thoreen C.C. et al (2012) A unifying model for mtorc1-mediated regulation of mRNA translation. Nature, 485, 109–113. 22552098 10.1038/nature11083 PMC3347774

[btw585-B10] Wolfe A.L. et al (2014) RNA g-quadruplexes cause eif4a-dependent oncogene translation in cancer. Nature, 513, 65–70. 25079319 10.1038/nature13485 PMC4492470

[btw585-B11] Zhong Y. et al (2015) Protein translational control and its contribution to oncogenesis revealed by computational methods. BMC Bioinformatics, 16, A6.

